# Mental health impairment and recovery after a road traffic injury: where do we stand in Europe?

**DOI:** 10.3389/fpubh.2024.1418920

**Published:** 2024-10-18

**Authors:** M. Papadakaki, B. Strukcinskiene, T. Alves, J. Lund

**Affiliations:** ^1^Laboratory of Health and Road Safety (LaHeRS), Department of Social Work, School of Health Sciences, Hellenic Mediterranean University, Heraklion, Greece; ^2^Injury Prevention and Safety Promotion Section, European Public Health Association, Utrecht, Netherlands; ^3^European Association for Injury Prevention and Safety Promotion, EuroSafe, Amsterdam, Netherlands; ^4^Faculty of Health Sciences, Klaipeda University, Klaipeda, Lithuania; ^5^Epidemiology Department, National Institute of Health Doutor Ricardo Jorge, Lisbon, Portugal; ^6^Norwegian Public Health Association, Oslo, Norway

**Keywords:** road traffic, injury, mental health, PTSD, depression, recovery, quality of life, disability

## Abstract

Individuals sustaining road traffic injuries (RTIs) have been shown to run an increased risk of impaired mental health over time and delayed recovery. It is often the case that mental health symptoms get less clinical attention among individuals sustaining RTIs and therefore psychological support tends to be delayed. Effective management of these aspects in a clinical setting is still challenging in Europe due to health systems’ unpreparedness to predict the risk of poor mental health outcomes among survivors and appropriately intervene. Although a considerable amount of research is available in Australia, Canada and the US, the problem is still under-investigated in Europe. This paper reports on a review of the literature, which aims at identifying and presenting the latest research on the predisposing risk factors of poor mental health recovery among individuals sustaining an RTI in Europe. The review identified a huge mental health burden remaining long after the road traffic incident and a complex interplay of factors affecting mental health recovery after an RTI. Several challenges have been identified including the lack of a consistent definition for mental health recovery, the use of heterogeneous instruments and non-consistent epidemiological approaches and the lack of data collection mechanisms in Europe to capture the true impact of injuries. The paper concludes that existing efforts to fully understand the mental health outcomes of RTI patients remain inconsistent in Europe and offers evidence-based solutions to guide public health research and policy.

## 1 Introduction

A consistent pattern of mental health outcomes have been recognized in literature among individuals sustaining a road traffic injury (RTI) along with an increased heterogeneity in recovery times (1, 2, 59, 60). In fact, evidence suggests that individuals suffering RTIs may differ in their recovery compared to other traumas in terms of symptoms’ onset, variability and chronicity. More precisely, survivors’ have been shown to run an increased risk of psychiatric disorder onset (3), frequent changes in their mental health status over time (4) and a high risk of symptoms’ chronicity (5). Even minor injuries have been shown to have chronic mental health consequences including reduced health-related quality of life and delayed return to work. In 2020 approximately 5.4 million of Europeans were treated in hospital Emergency Departments for RTIs (6), a substantial proportion of whom will develop a mental health condition post-crash. Despite the associated mental health burden, psychological aspects get less clinical attention as compared with the physical aspects of the injury and therefore psychological support tends to be delayed (7, 8). Effectively managing these issues in a clinical setting is still challenging due to the complex interplay of factors that need to be evaluated under a very busy schedule and a lack of knowledge and expertise (9, 10).

Despite the emerging evidence on the huge and enduring mental health burden, very few trajectories have been investigated in individuals sustaining RTIs (11, 12) and more research efforts have been warranted to identify modifiable risk factors in this population (13, 58). A critical need for more research has been stressed particularly in terms of minor-to-moderate injuries, where evidence is scarce due to underreporting (57). This is a pre-requisite for early identification of individuals at risk of prolonged mental health recovery and a critical step for early access to treatment (14, 15).

In response to this pressing need for additional efforts, a considerable amount of research has been initiated in Australia, Canada and the US during the last decade, toward examining RTIs and their mental health outcomes (5, 13, 16–18, 58) Survivors have been studied in these regions for up to 24-months and have been shown to suffer prolonged psychiatric morbidity at very high rates (almost 50%) (5). Hence, this is not the case for Europe, where research is limited even though RTIs are a major health problem and a leading cause of mortality and disability (19–22). In fact, there have been some collaborative projects aiming at investigating the burden of injuries either on mixed trauma populations or on specific injury types (e.g., Traumatic Brain Injuries, Spinal Cord Injuries), with RTIs remaining understudied. Comprehensive overviews of previous literature on recovery following RTIs have been published in two systematic reviews but evidence from Europe is scarce (10, 23). Therefore, the aim of this paper is to identify and present the latest literature on the predisposing risk factors of poor mental health recovery among individuals sustaining an RTI in Europe. More precisely, the paper reports on: (a) the mental health outcomes after an RTI, (b) the factors associated with mental health recovery after an RTI, and (c) the methodological limitations, research gaps and implications for future research, policy and practice.

## 2 Research strategy

This systematic review was conducted to explore the current literature on injuries sustained in road traffic crashes and the recovery process with emphasis placed on the mental health sequelae. WHO defines mental health as “a state of well-being in which an individual realizes his or her own abilities, can cope with the normal stresses of life, can work productively and is able to make a contribution to his or her community. The current study adopted “mental health” as an umbrella term for common post-crash mental health outcomes acting as predictors of long-term impairment, including post-traumatic stress disorder (PTSD), depression, panic disorder, generalized anxiety disorder, and substance use. A number of key terms were initially searched in PubMed, Scopus and Google Scholar. The search strategy included the following keywords; ‘injury’ AND ‘motor vehicle crash’ OR ‘traffic accident’ AND ‘recovery’ OR ‘disability’ AND ‘mental health.” Articles met the following criteria: published between 2014 and 2023; research papers; published in English language; European region as geographical area of reference. Exclusion criteria: studies not addressing RTIs but mixed-trauma populations, studies on injury mortality, and secondary publications such as opinion pieces. Articles reporting on the same study sample were excluded with those providing more detailed and complete methodological information on our research questions, retrieved for analysis [e.g., (24, 25)]. Reference lists were examined for additional evidence and “citation snowballing” was employed as a complementary process to primary search to ensure that studies, which are “hard-to-find” due to inconsistent use of terminology and reporting, are located and included in the review. Two researchers read abstracts of each retrieved article to determine eligibility.

## 3 Quality appraisal and synthesis

All the eligible full-text papers were retrieved and screened by two independent reviewers and critically appraised for the quality of the evidence and risk of bias using the Newcastle-Ottawa Scale (NOS) for cohort studies, which considers the case definition, participants’ selection, comparability of study groups, exposure and outcome data to calculate a score, based on the reliability of the data. A NOS score of ≥ 6 indicates high quality, with a maximum total score of 9. For the outcome subcategory, a minimum duration of 3 months after the crash was set while for the subcategory of adequacy of follow-up was set to 50 per cent. Other study limitations were considered with particular emphasis placed on the coherent conceptualization of study constructs, the adequacy of study designs and the level of methodological soundness. No studies were excluded based on the quality criteria though the appraisal identified inadequate descriptions of study parameters and risks of bias (see Table 1). Based on the assessment, the overall quality was below the threshold. All the studies confirmed the RTI via medical records and described those lost to follow-up. Common limitations were the lack of non-exposed subjects, poor baseline assessment of mental health state prior to the crash (e.g., self-reported, retrospective or short-term), use of self-reports to assess the outcome and incomplete follow-up.

**Table 1:** Characteristics of the four selected studies.

Authors	Design	Purpose	Setting / Population	Mental health outcomes at post-injury	Risk factors of poor mental health recovery	Study limitations and risk of bias*
Nhac-Vu et al. (42)	Prospective study / follow up at 12 months following the RTC	To identify predictive factors of patients’ outcomes 1-year post RTCs	All Hospital Units Rhone administrative Department of France /616 road crash victims in France	Rate of PTSD at 1 year (19%)	Age > 24 years, initial injury severity, injury type (spinal or lower limb injuries), socio-economic fragility, involvement of a relative in the accident.	Incoherent conceptualization of recovery and mental health constructs; Weak justification of measurements’ selection; Weak framework of analysis due to missing data at follow up, small sample sizes and low statistical power; Mental health outcomes reported for small groups (NOS = 4)*.
Dooh an et al. (24)	Mixed method study (qualitative/quantitative) / follow up 1 to 3 months following the RTC	To explore physical and mental consequences and injury mechanisms among bus crash survivors and identify aspects that influence recovery.	Swedish Accident Investigation Authority (SAIA; Stockholm, Sweden), Post-crash investigation /56 survivors from a bus crash in Sweden	17 (31%) had a high risk (TSQ ≥ 6) for PTSD.	Higher mental distress among survivors living with moderate to severe physical injuries or with a partner who sustained moderate to severe injuries.	Weak epistemological orientation; Incoherent conceptualization of recovery and mental health constructs; Weak justification of measurements’ selection; Short follow up period; Non-validated research instrument; Non-validated framework of combined analysis of mixed method data (NOS = 2)*.
Papadakaki et al. (21, 43)	Prospective study / follow up at 1–6-12 months following the RTC	To examine the psychological and physical consequences of injuries sustained in road traffic crashes in a group of road crash survivors 6 and 12 months after the injury.	7 Hospital Intensive Care Units (ICU) in 3 Countries / 239 road crash victims in Greece, Germany, and Italy	*At 6 months post-injury: 39.6% PTSD, 33.0% Depression. *At 12-month post-injury: 21.1% PTSD, 23.3% Depression *Lower risk of Depression: 79% at 6-months and 88% at one-year. *Lower risk of PTSD: 72% at one-year.	Injury severity (higher scores), injury type (lower limb injury), initial psychological response (higher distress immediately after the injury), age (older), user type (cyclists, pedestrians).	Incoherent conceptualization of mental health constructs; Weak justification of measurements’ selection; Mental health state not assessed at pre-injury level; High drop-out rate in one study site (NOS = 5)*.
Kova cevic et al. (20)	Prospective study / follow up at one-month post-injury	To evaluate the quality of life of the RTA survivors and identify factors associated with decreased quality of life after the RTA.	Institute of Emergency Medicine in one County of Croatia/ 200 RTA survivors with and without injuries in Croatia	35.5% (PTSD) 20.0% (Depression) 4.5% (Anxiety)	*Reverse correlation of mental health outcomes with all QoL domains after the RTA. *Mental health after RTI associated with age, self-assessed economic status, poor pre-RTA health (chronic disease, psychiatric disease, previous permanent pain, use of medications), injury-related factors (injury affliction, injury severity self-assessed life-threat pain following the RTA).	Incoherent conceptualization of mental health constructs; Weak justification of measurements’ selection; Participant recruitment process not detailed; Short follow up period; Weak assessment of mental health state at pre-injury level; Participants’ performance/ mental health outcomes not reported (NOS = 4)*.

*NOS, Newcastle-Ottawa Scale score.

All studies were then summarized in Table 1 with the following headings: authors; design; purpose; setting/population; mental health outcomes at post-injury; risk factors of poor mental health recovery; study limitations and risk of bias. Decisions about which data to be extracted from individual studies were guided by the review objectives. Meta-analysis was not considered for this review because of the low quality of the identified studies. Meta-analyses would be performed only if more than three studies were above the quality assessment threshold. Therefore, to facilitate interpretation of evidence, we used descriptive information. A narrative summary was used to describe the included studies and their findings, while enabling the identification of patterns across the studies as well as the exploration of relationships within and between studies, based on commonalities in outcomes, study designs and instruments used across the identified studies.

## 4 Results

### 4.1 Description of available studies from Europe

**Figure 1:**
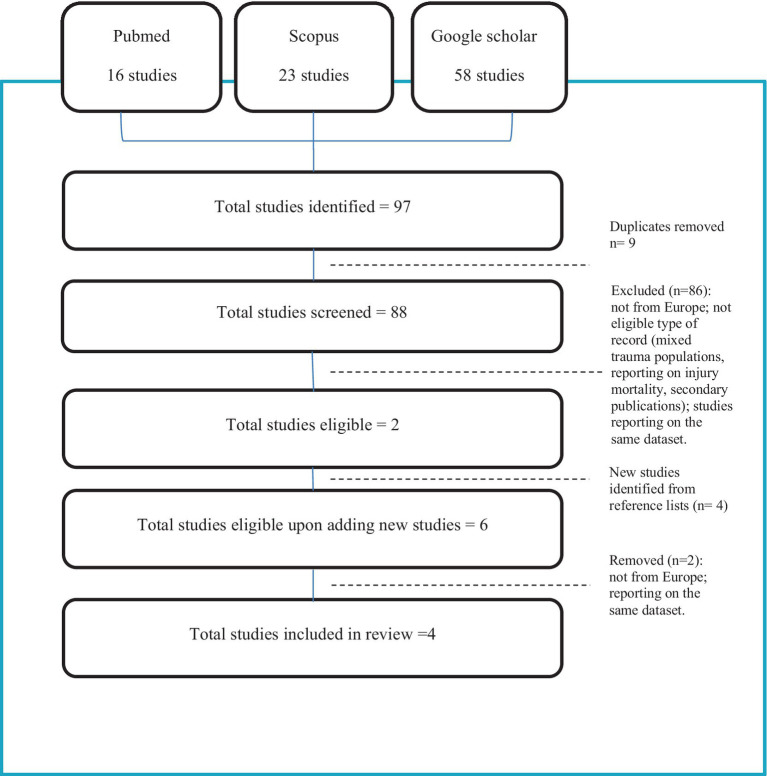
PRISMA flow diagram.

A total of 97 articles (23 Scopus, 16 Pubmed, 58 google scholar) were identified (88 unique citations after the removal of duplicates; 2 retrieved for analysis). Four more articles were identified through the review of the reference lists of the eligible articles (3 eligible, 1 removed due to reporting on the same study sample; 2 retrieved for analysis). Four articles in total were retrieved for analysis (see Figure 1 flow diagram).

Studies that were excluded from the analysis were primarily from countries outside of the European region such as Australia [e.g., (2, 13, 26)], United States [e.g., (27, 28)], Canada [e.g., (29, 30)], Asia (31, 32) and Africa [e.g., (33)]. Studies from the European region derived primarily from the Netherlands and Norway and most often were excluded from the analysis due to investigating mixed trauma populations [e.g., (34, 35)] or due to focusing on specific injury types [e.g., (36–38); focus on Traumatic Brain Injuries / (39, 40); focus on Traumatic Spinal Cord Injuries]. Studies from Europe aimed at describing the prevalence and prognostic factors of mental health symptoms, quality of life as well as functional and psychological recovery after injury in clinical trauma populations [e.g., (34, 37, 40)]. They most often employed Emergency Departments (EDs) or Intensive Care Units (ICUs) encounters [e.g., (34, 37, 41)], with the follow up ranging from 6 months [e.g., (38)] to 5 years post-injury [e.g., (36)].

As for the studies that were retrieved for analysis, except for the study of Doohan et al. (24), which constitutes a post-crash investigation of 56 survivors, by a national Swedish Authority, the three remaining studies employed prospective research designs to measure a wide range of physical, psychological and functional outcomes following an RTI with medium sample sizes (ranging from 200 to 886 survivors). The settings in these studies were either Emergency Departments (EDs) (20, 42) or Intensive Care Units (ICUs) (21, 43, 44) and some of the studies focused on specific hospitals while others reported county-wide data. Depending on the study setting, populations differed in terms of injury severity scores with serious or critically injured patients represented more in one study than others (21, 43). Follow up ranged from one-month (20, 24) to a maximum of one-year post-injury (21, 42, 43). Health-related quality of life was used in all the prospective studies as a key concept to measure the “recovery process” of the RTI survivors with mental health captured as one of the multiple dimensions of quality of life. Self-reported symptoms of post-traumatic stress disorder (PTSD), depression and anxiety were also evaluated as indicators of mental health comorbidity after an RTI.

### 4.2 Prevalence of mental health outcomes after an RTI

Symptoms of PTSD and depression were the most consistently reported mental health outcomes. PTSD symptoms were reported by 35.5% of survivors one-month post-injury, by 39.6% at 6 months and by 19.0–21.1% at one-year post-injury. Likewise, depression was reported by 20.0% one-month post-injury, by 33.0% at 6 months and by 23.3% at one-year post-injury. At one-year post-injury, there was an 88% lower risk of depression and a 72% lower risk of PTSD.

### 4.3 Factors shown to contribute to poor mental health recovery after an RTI

A diverse range of factors were shown to be associated with mental health morbidity following RTIs, including socioeconomic factors, pre-injury health, injury-related factors and other incident-related circumstances. Many of these factors have already been identified in previous systematic reviews (10, 23). As regards to the socioeconomic factors, age and self-assessed economic status had a strong effect on mental health recovery after an RTI (20, 21, 42, 43). In Papadakaki et al. (21) the risk of depression at one-year post-injury increased by 5% with every additional year of age and in Nhac-Vu et al. (42) age > 24 years was predictive of poor outcome at 1 year. Papadakaki et al. (21) identified a 7.49 times higher risk of survivors being depressed at 6 months post-injury, if divorced or widowed as compared with single. Nhac-Vu et al. (42) identified increased risk of poor outcomes at one-year post-injury among individuals who lived alone, resided in disadvantaged areas, had low educational attainment and occupational instability or lacked health insurance to address their health care needs. As for the impact of pre-injury health, pre-existing chronic disease, psychiatric disease and pain as well as the use of medication before the RTI, increased the risk of enduring psychological impairment after an RTI (20, 21). Injury-related factors linked with poor mental health outcomes were injury severity, suffering and pain severity (20), body region injured (low-limb injuries had poor outcomes at one-year post-injury) (21, 42), initial psychological reaction (those who developed depression immediately after the injury had 4.77 times higher risk of being depressed at 6 months post-injury and 4.81 times higher risk at 12 months post-injury) (21). As for incident-related circumstances, Nhac-Vu et al. (42) and Doohan et al. (24) found that the involvement of a relative in the incident had a major effect on the recovery process, with survivors’ well-being being directly affected by their family’s well-being. Moreover, vulnerable road users (e.g., pedestrians, cyclists) were shown to be more prone to poor mental health outcomes as compared with other road users. In Papadakaki et al. (21) four-wheel users had 85% decreased risk of developing depression at 12 months post-injury as compared with pedestrians and cyclists.

## 5 Discussion

What clearly comes out of this review is the fact that despite the growing interest in mental health outcomes after an RTI, research is still limited in Europe. The few available studies identified in this review have several methodological limitations (see Table 1) related to their study design (e.g., inconsistent conceptualization of the mental health target and outcomes, short follow up periods limited to 12 months), their participant recruitment techniques (e.g., poor description or non-probabilistic recruitment), and the measurements used (e.g., inconsistent selection of tools and instruments to evaluate the outcomes). More epidemiological studies are needed in Europe with longitudinal study designs and longer follow-up periods to allow for the exploration of these complex trajectories and factors that influence recovery. Non-injury healthy controls could be included for improved research outcomes on psychiatric comorbidity in order to address non-RTI related confounding factors (15, 45).

Another issue clearly identified in this study is the lack of a consistent definition for mental health recovery after an RTI, which is thought to result in the use of heterogeneous instruments and non-consistent epidemiological approaches (see Table 2). In general, recovery after an RTI seems to lack a standardized definition (10), with part of the literature assuming recovery based on improved performance in quality of life measures, mental health status, return to work, disability levels, while another part of the literature employing definitions of recovery, which are guided by regulatory authorities and are based on the status of the compensable injury (23, 46). In this review, we realize that studies employ a narrow clinical definition of recovery, which emphasizes one’s psychiatric symptoms and functioning without encompassing psychological aspects such as resilience, coping, self-efficacy and spirituality and without taking into account one’s attitudes, feelings, goals, and skills to live within the limitations caused by the injury. We have also noticed that specific mental health conditions have been repeatedly selected as indicators of mental health recovery after an RTI (e.g., PTSD, depression, anxiety, health-related quality of life) and a variety of instruments have been employed to measure the degree of impairment over time (*Trauma Screening Questionnaire, TSQ*; *Impact of Event Scale, IES-R; Center for Epidemiological Studies Depression Scale, CES-D; PTSD Checklist for Civilians, PCL-C; Beck Anxiety Inventory, BAI; Beck Depression Inventory, BDI-I; WHOQoL-bref for HRQoL; SF-36; WHODAS II*). This inconsistency in epidemiological research has been thought to strongly affect comparability of data and potentially hinder the establishment of screening criteria for poor mental health recovery. In light of these limitations, a universal definition of recovery after an RTI, has been seen as critical for improved understanding of risk factors of poor recovery as well as improved identification and treatment of those at risk (22, 47, 48). Considering the variety of definitions and instruments used interchangeably and the mixed results, some studies have endorsed the use of quality-adjusted life year (QALY) (49) and the disability-adjusted life year (DALY) (50) as measures of injury burden that could potentially promote comparability among study outcomes. Berg et al. (51) also proposed the Risk of Permanent Medical Impairment (RPMI) concept (52) and the Function Capacity Index (FCI) (53) as benchmarks of medical disability to enable comparisons of long-term consequences of injuries among European countries. However, no consensus has been reached yet on the methods that best capture these complex aspects of recovery in the long run.

**Table 2:** Outcome measures and instruments used for mental health recovery after an RTI.

Authors	Mental health outcomes to measure recovery	Instrument used to measure recovery	Study limitations and risk of bias
Nhac-Vu et al. (42)	Post-Traumatic Stress Disorder (PTSD)	Post Traumatic Stress Disorder Checklist Scale (PCLS)	17 items assessing re-experiencing (items 1–5), avoidance (items 6–12) and increased arousal (items 13–17). Responses anchored from “1 = not at all” to “5 = very often.” The threshold of 44 was applied to indicate possible PTSD.
	Mental health as a dimension of general health.	World Health Organization Quality of Life Assessment (WHOQOL-BREF)	26 items: 2 items assessing overall satisfaction with life and general sense of personal well-being and 24 items assessing 4 domains: physical health (7 items), psychological health (6 items), social relationships (3 items), and environment (8 items). Responses anchored from 1 to 5, summed, and transformed into a scale from 0 (worst health- related quality of life) to 100 (best health-related quality of life).
(Dooh an et al. (24))	Post-Traumatic Stress Disorder (PTSD)	Trauma Screening Questionnaire (TSQ).	10 items assessing PTSD risk after potentially traumatic experiences. Items covered two of the PTSD criteria: re-experiencing and arousal symptoms. “yes” or “no” responses. Six or more positive answers indicated risk of developing PTSD.
Papadakaki et al. (21, 43)	Post-Traumatic Stress Disorder (PTSD)	Impact of Event Scale (IES-R)	15 items assessing PTSD risk. Two subscales; the “Intrusion Scale” (7 items) and the “Avoidance Scale” (8 items). Responses anchored from “0 = not at all” to “5 = often.” Higher scores indicated greater stress symptoms.
	Depression	Center for Epidemiological Studies Depression Scale (CES-D Scale)	20 items assessing depressive symptoms over the previous week. Responses anchored from 0 to 3 (0 = Rarely or none of the time, 3 = Most or all the time). Four items were worded positively and reverse coded. Higher scores indicated greater depressive symptoms.

Despite the above-mentioned methodological challenges, a huge mental health burden has been identified in this review with symptoms of depression, PTSD and anxiety remaining long after the road traffic incident. The recovery trajectory seems to vary widely with a large percentage of survivors in need of extensive time periods for full recovery (5, 13, 18, 58). Given the high incidence of mental health impairment among RTI survivors, is seems essential to ensure that mental health concerns are addressed alongside physical injuries at all levels of health care. Implementing predictive screening at the location of the incident and during initial medical assessments is critical for those at risk of sustaining long-term mental health impairment. Likewise, ensuring access to psychological counseling and trauma-informed care as well as anticipating professional assistance in the process of psychological adjustment to the acquired disabilities, is critical for patients’ recovery. Most importantly, mental health assessment and individually tailored interventions need to be integrated into the standard care protocols for RTI patients to ensure an efficient health system’s response.

What stands out of this review, is the complex interplay of factors affecting mental health recovery after an RTI. Mental health resilience following RTIs is better understood upon considering a variety of factors related to the individual, the injury and the incident. In our study, the injury type (lower limb injury), initial psychological response to the injury (higher distress immediately after the injury), user type (cyclists, pedestrians), pre-existing physical or mental health problems, socioeconomic fragility and performance in various “Quality of Life” domains (lower scores in various domains including physical health, social functioning, etc), were common factors that influenced the risk of poor mental health recovery after an RTI. Most of these factors are already known from previous research from countries outside Europe (13, 15, 54). This observation implies that there is no silver-bullet solution to prevent poor mental health recovery among RTI survivors.

Interestingly, the current study generates important evidence on the impact of socioeconomic factors on mental health recovery among RTI survivors. It is often the case in research to place emphasis on the physical disability and the functional independence of the individuals and overlooks the capacity of a person to continue functioning. Multiple studies indicate that low-income and low SES households lack access to resources that they need after traumatic events (55). Changes in the employment position or the salary, in-house adaptations, childcare arrangements and the need for paid child-caregiver are often “neglected” parameters after an RTI, which constitute a huge burden for low-income families. Despite this fact, we realized that social, financial, and familial consequences are rarely investigated in the literature on RTIs, most probably due to a lack of investment in this domain and also due to difficulty in accessing such information from public registries. Most importantly, expertise in economic estimation of social capital in this research and policy domain is still low in many EU countries. Assessing all this information, could on one hand offer an opportunity of a holistic assessment of the circumstances caused by the traumatic event on individuals’ lives and accurate interpretation of evidence, and on the other hand allow evidence-based decisions on the appropriate therapeutic solutions upon considering the social capital of the individuals (43).

### 5.1 Study limitations

A number of limitations have been identified and need to be acknowledged. The current review included only three databases and this implies that there may be other studies not captured in this review. We only included studies written in English language and we may have missed findings reported in other languages. Citation snowballing, although useful in detecting “hard-to-find” studies, it should be used with caution due to being susceptible to selection biases. The study employed “mental health” as an umbrella term to capture the state of participants’ well-being. It is possible that there are studies focusing on specific mental health conditions or outcomes that may fall within the scope of the study but not identified in this review. Critical appraisal of identified studies did not explicitly inform the synthesis stage, and therefore did not influence the review outcomes. This implies that the review findings may be biased due to including studies with low quality and internal validity. Likewise, the small sample of identified studies and their diverse methodological characteristics made it inappropriate to undertake a meta-analysis or infer that the findings can be generalized to other EU countries. More longitudinal studies are thus warranted in the future to facilitate interpretation of the complex mental health recovery trajectories after injury and improve our understanding of how subgroups adjust following an RTI. Lastly, given the methodological limitations and the research gaps revealed in this review, based on insights from articles focusing on Europe, a higher level of inclusion could be considered as useful and more impactful in future systematic reviews on the mental health recovery of RTI survivors.

## 6 Conclusion

Efforts to fully understand the mental health outcomes of patients sustaining RTIs, remain inconsistent in Europe. There are few challenges to be mentioned. First, injury data collection and analysis are still problematic in Europe. Many countries still lack the data and the systems for collecting accurate and comprehensive information on the burden of RTIs and mental health outcomes, and this makes it difficult to understand the true impact of injuries on populations and guide public health interventions. Second, even the few countries in Europe that have more advanced injury registries and robust data management systems (e.g., Norway, the Netherlands, Belgium) are still struggling with data linkage challenges, inconsistent injury coding systems, missing data due to non-mandatory recording, privacy and security concerns. The complex mechanism of injuries cannot be understood if access to valid data is not granted, and countries in Europe still lack a comprehensive picture of morbidity due to injuries and their predisposing factors. The EU-IDB (European Injury Database), operated under the European Association for Injury Prevention and Safety Promotion (EuroSafe) and the European Burden of Disease Network of the WHO Regional Office for Europe are leading initiatives in Europe, currently acting as a technical platform for integrating and strengthening capacity in the assessment of injury burden across Europe. Apart from this, it is critical for Europe to invest more efforts on systematic collection of data on risk exposures, better diagnostic tools and prediction models of mental health morbidity to accurately predict mental health outcomes. This can be achieved through equipping hospitals with standardized assessment tools, clinical evaluation protocols and trained mental health professionals to early address risk factors and facilitate a successful mental health recovery after an RTI. In fact, a modern comprehensive trauma system should start with injury recognition, continue with triage to a trauma center, multidisciplinary inpatient care, and outpatient follow-up of long-term physical and psychosocial sequelae (56). To manage this stepwise process, it is critical for health care systems to run gap analyses and develop action plans. Most importantly, it is critical for systems to select performance measures, establish collaborative relationships and operational processes as well as adopt a core set of trauma-related skills to optimize medical and nursing post-injury care. The American College of Surgeons (ACS) released new guidelines in 2023 to assist trauma centers in efficiently addressing mental health issues among patients who have experienced a traumatic injury. Investment on interprofessional education and joint curricula, is also critical as it will allow a holistic understanding of patient care, emphasizing the importance of addressing not only the physical but also the psychological and social aspects of recovery. Addressing patients’ needs holistically upon hospital discharge, will strengthen personalized care and will support patients in building resilience and coping strategies. Most importantly, managing service integration between health (medical, psychological) and social services (rehabilitation, community support) will improve patients’ access to information and will ensure continuity of care, which is essential for recovery. In fact, collaboration with social services can provide patients with access to resources like counseling, financial support, and community programs while offering a support network, education and support groups, which are vital for mental health recovery. What is even most important is securing a strong political commitment to prioritize injury prevention efforts among other topics in the political agenda. This requires synergies, joint policy-making, aligned goals among different sectors and high public acceptability. To make it feasible, it is necessary to ensure financial and technical resources, a legal mandate and “a champion” at higher political levels to drive implementation.
